# Building an Equity-Centered Ecosystem: University of Utah Health as a Microcosm

**DOI:** 10.1007/s40615-024-01982-6

**Published:** 2024-04-04

**Authors:** Quang-Tuyen Nguyen, Victoria Cabal, Michelle Debbink, David Acosta, Valerie J. Flattes, Donna Baluchi, Natasha Ovuoba, Paloma F. Cariello, Bart T. Watts, Erin R. Clouse, Heather Nyman, Eliza Taylor, Line Kemeyou, Julie E. Lucero, Judy C. Washington, Edgar Figueroa, Kendall M. Campbell, Abdulkhaliq Barbaar, Stacy A. Ogbeide, José E. Rodríguez

**Affiliations:** 1 Department of Pediatrics, Spencer Fox Eccles School of Medicine at the University of Utah, Salt Lake City, UT USA; 2 Rankin Climate, LLC, Salt Lake City, UT USA; 3 Department of Obstetrics and Gynecology, Spencer Fox Eccles School of Medicine at the University of Utah, Salt Lake City, UT USA; 4 Association of American Medical Colleges, Washington, DC USA; 5 College of Nursing, University of Utah, Salt Lake City, UT USA; 6 Eccles Health Sciences Library, University of Utah, Salt Lake City, UT USA; 7 Huntsman Cancer Insitute, University of Utah, Salt Lake City, UT USA; 8 Department of Internal Medicine, Spencer Fox Eccles School of Medicine at the University of Utah, Salt Lake City, UT USA; 9 School of Dentistry, University of Utah, Salt Lake City, UT USA; 10 University of Utah Medical Group, Salt Lake City, UT USA; 11 Department of Pharmacotherapy, College of Pharmacy, University of Utah, Salt Lake City, UT USA; 12 Department of Family and Preventive Medicine, Spencer Fox Eccles School of Medicine at the University of Utah, Salt Lake City, UT USA; 13 Department of Health and Kinesiology, College of Health, University of Utah, Salt Lake City, UT USA; 14 Atlantic Health, Morristown, NJ USA; 15 Weill Cornell Medicine, New York, NY USA; 16 Department of Family Medicine, University of Texas Medical Branch, Galveston, TX USA; 17 University of Utah Health Equity, Diversity and Inclusion, University of Utah, Salt Lake City, UT USA; 18 Department of Family Medicine, University of Texas Health Sciences Center, San Antonio, TX USA

**Keywords:** Underrepresented in medicine, Diversity equity inclusion, Medical education, Pathway, Pipeline programs

## Abstract

Academic medicine, and medicine in general, are less diverse than the general patient population. Family Medicine, while still lagging behind the general population, has the most diversity in leadership and in the specialty in general, and continues to lead in this effort, with 16.7% of chairs identifying as underrepresented in medicine. Historical and current systematic marginalization of Black or African American, Latina/e/o/x, Hispanic or of Spanish Origin (LHS), American Indian/Alaska Native, Native Hawaiian/Pacific Islander, and Southeast Asian individuals has created severe underrepresentation within health sciences professions. Over the last 30 years, the percentage of faculty from these groups has increased from 7 to 9% in allopathic academic medicine, with similar increases in Osteopathic Medicine, Dentistry, and Pharmacy, but all lag behind age-adjusted population means. Traditionally, diversity efforts have focused on increasing pathway programs to address this widening disparity. While pathway programs are a good start, they are only a portion of what is needed to create lasting change in the diversity of the medical profession as well as the career trajectory and success of underrepresented in medicine (URiM) health professionals toward self-actualization and positions of leadership. This article elucidates all parts of an ecosystem necessary to ensure that equity, diversity, and inclusion outcomes can improve.

## Introduction

**Fig. 1:**
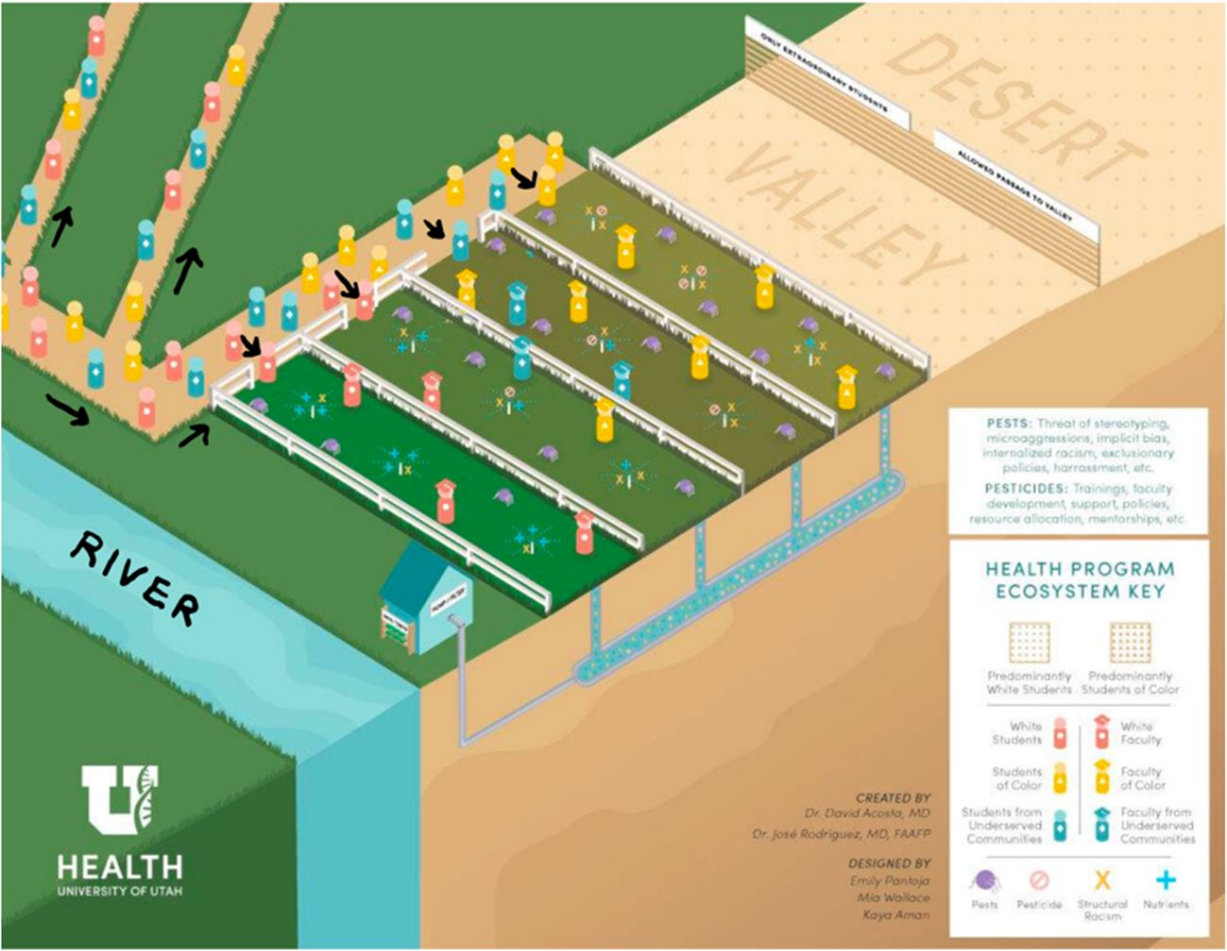
The equity-centered ecosystem graphic

In consultation with diversity leaders across the country, Dr. David Acosta first presented the Health Systems Ecosystem model (Fig. 1) of an equity ecosystem to multiple cohorts of “Healthcare Executive Diversity and Inclusion Certificate” participants at the Association of American Medical Colleges. Dr. Acosta drew this diagram. Emily Pantoja, Mia Wallace, and Kaya Aman, graphic designers for the University of Utah Equity, Diversity, and Inclusion division, created the graphic illustration of Dr. Acosta’s sketch. This manuscript is designed to highlight the multiple intersections of equity, diversity, and inclusion work, not unlike Dr Camara Jones’ work on racism, using the metaphor of a garden and a gardener [1]. This adds to the literature in two fronts:

1. This is the first description of the ecosystem necessary for success in EDI in the academic health sciences literature.
2. This paper illustrates the many areas where EDI work is needed to ensure success in increasing the proportion of underrepresented students, residents, faculty, and staff working in health care.

EDI work does not survive when only parts of the ecosystem are supported. Success depends on the entire ecosystem working together. In addition, this paper provides tools and strategies to build this ecosystem.

## Description of the Model Components

This equity ecosystem is based on a metaphor taken from farming. To get good fruits or vegetables, you need multiple components—good soil, water, fertilizer, and pesticides [2]. We begin by explaining what each farming component represents, and state how it fits into academic health sciences equity work.

In the center of the graphic there are four garden plots, each fenced in, some with larger openings than others, and some with closer proximity to the water. These plots represent the different health sciences schools. In most academic health centers, the program with the most resources is a medical doctor program at the school of medicine. At University of Utah Health, that is the case, and the Spencer Fox Eccles School of Medicine has the bulk of the resources along with the EDI infrastructure of Health Sciences. The School of Medicine has funded an Associate Vice President for Health Equity, Diversity, and Inclusion, [3] as well as an Associate Dean for Health Equity, Diversity and Inclusion [4] for the past 15 years, while no other diversity offices existed at other schools. In most academic health centers, the program with the most resources is the medical doctor program at the school of medicine. For these reasons, the example that has the greenest grass and the plot closest to the river is the school of medicine.

There are three other plots that can represent any of the other health sciences schools. Each of them is independent, providing professionals to work in the hospital, pharmacy, physical and occupational therapy, and dental and nursing disciplines, as the University of Utah uses a Responsibility Center Management (RCM) Model. As the school of medicine generates the highest revenue, they receive the most resources. Other schools are also dependent on the school of medicine for that reason. Additional funds generated through clinical service or research can be used to fund initiatives outside of the school of medicine. Because of its stature, the school of medicine does lead the charge toward equity, and the AVP has always been a faculty member at the school of medicine, and the revenues from medical care continue to advantage the medical school.

In the graphic, the relationship between health professions schools is manifested in how they get water. Water goes from the river to the school of medicine plot, and then to the other plots. There are great inequities in the educational space for health professionals, and University of Utah Health is working toward change. Through intentional collaboration, we are using pathway programs originally designed only for MD program students and adapting them to serve all the pre-health sciences students.

While the pathways that lead to the garden plots are means for students to get into health sciences professional schools, the whole ecosystem needs to be supported. For decades, the work of equity, diversity, and inclusion has focused almost exclusively on these pathway programs. University of Utah Health has multiple pathway programs which have documented their success through medical literature. They are the Native American Summer Research Institute (NARI), [5] the Path Maker program,[6] the Physician Assistant program, [7] the Family Medicine Residency program, [8, 9] Health Sciences LEAP (Learning, Engagement, Achievement and Progress), [10–12] and the Saturday Academy program, [13] all of which are funded with school of medicine grants or monies generated clinically. Each one of these programs has published their diversity successes in the medical literature. Yet, even with documented success in this space, we are moving more slowly toward significant change in the faculty composition and leadership of health sciences partially because all efforts were concentrated to the pathway programs, while all areas of the ecosystem needed attention. Even the successful pathway programs had learners leaving on side paths that took them away from graduate studies in the health sciences. This effect is even more profound when you look at underrepresented in medicine students (URiM) as defined above. While only a small percentage of those who enter the pathway to health sciences graduate education, they leave the pathway in greater numbers than their non-URiM peers. This has many reasons, but one of them is the atmosphere and ecosystem of our graduate education programs.

The essential components of the garden ecosystem are soil, water, and nutrients. Without these, no garden can grow, and without their counterparts in academic medicine, equity efforts will fall short (Table 1). Like any garden, equity requires a strong foundation. This category can be divided into the basics needed for a healthy ecosystem: *Soil*, which provide organic material for plants to grow; *Water*, which nourish plants; and *Nutrients*, which improve soil and enhance plants.

**Table 1:** Growth-promoting components of the equity ecosystem

Component	
Soil	Soil provides the minerals and organic material necessary for crops to grow. The soil in the health science ecosystem is the existing infrastructure of the health sciences schools. Some schools or colleges (School of Medicine) have extensive endowments, large scholarships, and clinical income; others have less resources (College of Nursing or Health) and may wholly depend on tuition. Like in a garden, nutrient-rich soil can produce thriving fruits and vegetables, and in health science schools strong (nutrient-rich) infrastructure can produce more access
Water	Water within the ecosystem may be more consistent and reliable, allowing a steady measured stream of nurturance. Within the Health System Ecosystem model, water can represent grant-funded science that supports all health professionals (through indirect costs) or clinical income that can be used to support the academic mission of the health sciences campus
Nutrients	***Fertilizer: ***Fertilizer makes the soil better and provides essential growth enhancers to the plants. Those schools with more robust infrastructure have more opportunities to form mentoring relationships for students ***Mulch:*** Mulch is the material spread around or over a plant to insulate the soil, suppress weeds, improve moisture retention, improve fertility, and enhance visual appeal. Financial aid is the equivalent to mulch in the ecosystem analogy and ensures that students do not need to work during health professions training ***Diversity of crops:*** When different crops are planted in proximity, they add nutrients, such as when legumes are planted with corn [14]

Like any garden, plants can be harmed by pests, harmful microorganisms, weeds, and varying ground conditions. It is important to not only understand essential farming components, but the stunting components as well. In the equity ecosystem, the stunting components manifest as biases, racism, stereotype threat, and harassment (Table 2). Like any garden, equity efforts can be derailed by hate and discrimination (pests). This category can be divided into *Weeds*, which compete for water and nutrients; *Insects*, which slowly bite at plants, taking small pieces at a time; *Microorganisms*, which infect and kill plants from the inside; and *Animals*, which eat off large parts of plants and impede growth.

**Table 2:** Stunting components of the equity ecosystem

Weeds, insects, microorganisms, and animals
***Exclusionary policies:*** In a garden, weeds steal water and nutrients from the soil. In the same way exclusionary policies steal resources and opportunities away from others at the benefit of some. There are a variety of exclusionary practices within health sciences education, some of which have been recently legislated. Cliques of clinical professionals will cause feelings of intimidation where they are present. Lack of mentors, inefficient training, stereotyping, and other factors that work against a sense of belonging all contribute to exclusionary practices in health sciences education
***Microaggressions: ***Microaggressions chip away at individuals one aggression at a time. Microaggressions particularly affect historically excluded students as marginalized groups are most often the receivers of micro-assaults, insults, and invalidations
***Stereotype threat:*** The theory posits that this threat leads to underperformance and eventually avoidance of threatening situations. This threat of stereotyping, if left unchecked in health sciences education, will prevent diversity from flourishing within this ecosystem
***Internalized racism:*** Internalized racism hurts historically excluded health professionals from the inside. Internalized racism is the assimilation of negative societal views and stereotypes of one’s own racial/ethnic group
***Unconscious or implicit bias:*** Similarly, unconscious or implicit bias is an internal belief that can have negative effects on historically excluded learners and teachers. Regardless of intention, unconscious biases hurt historically minoritized and marginalized learners and faculty, and universities across the world are working on the elimination of this form of bias
***Harassment:*** In the form of aggressive pressure or intimidation, harassment can shut down equity efforts and inflict deep and immediate harm on the victim. Historically excluded students, women and others are more likely to be the recipients of direct harassment, thus shutting down

Like any garden, equity efforts can be cultivated by prevention and intervention efforts. This category can be divided into *Cultural controls*, which focus on changing the environment to redirect pests; *Mechanical controls*, which use physical objects to remove pests; *Biological controls*, which introduce plants or insects that control the introduction of pests into the garden; and *Chemical controls*, or poisons that stop pests. Periodic and consistent *Soil testing* offers important feedback on the status of the ecosystem and the various plants within it. Soil tests help ensure system maintenance for all plants to thrive (Table 3).

**Table 3:** Tending to the equity ecosystem

Tending component	
Cultural controls	***Systemic belonging:*** Changing the environment of the garden can ensure that nourishing insects and animals abound and pests are redirected outside the garden. In the same way, cultivating an inclusion health sciences school culture can ensure everyone thrives. Belonging is the goal of equity, diversity, and inclusion work. Belonging requires a culture that invites nourishment and redirects harmful behaviors, actions, and influences
Mechanical controls	***Accessibility measures (remove barriers):*** Mechanical controls use physical barriers or introduction of objects that remove pests through barricades or traps. In health sciences, sharing resources to offer more financial, intellectual, and physical access across health sciences schools will aid in removing barriers so that all individuals can thrive
Biological controls	***Training:*** Biological controls introduce healthy plants and insects that deter the introduction of pests into a garden. Similarly, instruction can be helpful for individuals and groups to develop through shared learning how to nourish the organization and thus remove the behaviors that impede equity and inclusion
	***Faculty development:*** In the same way, development focused on faculty mentorship and engagement can help health sciences schools ensure faculty have the necessary skills to remove harmful behaviors in the learning environment
Chemical controls	***Accountability measures (stop or reduce incidents of bias):*** Chemical controls center on stopping pests. Similarly, accountability creates processes and systems intended to stop or reduce incidents of bias and other harmful behavior. Accountability is about constantly checking the work against a set of questions: Who is benefiting and who is not? How can efforts be made to ensure everyone benefits?
Soil tests	***Climate surveys:*** Like a soil test that offers feedback on the health of the garden, climate surveys are designed to measure how employees feel about an organization

### Cultivating an Equity-Centered Gardener

The gardener represents the conscious ability to control variables within the ecosystem and, like Camara Jones’ allegory, The Gardener’s Tale, can control factors such as the fertility and health of the soil, or location of a particular plant and its access to water and sun. In Dr. Jones’ allegory, a gardener favoring a particular plant and not investing in equity could end up feeding an original bias by systematically favoring and protecting one plant over another. If the gardener is neglectful or uninformed, they can make mistakes that can be passed on and assumed instead to be due to the failure of a plant. An example of this may be a succulent that cannot tolerate excessive watering and will be at risk of rot. An attentive, informed gardener would be able to notice the change in vigor and adjust sun and water exposure appropriately; an inattentive, uninformed gardener may conclude the plant to be a poor specimen and pull the plant from the garden and search for a fresh, new specimen to take its place. Even worse, this gardener may develop partiality following these experiences and conclude that the succulent never belonged in the garden anyway, are delicate and fickle, no longer worth investment, and incapable of growing well in an established ecosystem without questioning why generations upon generations of plants have failed under the same systems of care. This gardener misses the diversity and representation in the garden, but also the resilience and potential role this water-wise plant may bring to the entire landscape in seasons of drought. Similarly, the Health System Ecosystem can only achieve equity when the faculty, staff, and administration (gardeners) tend to each learner (plant) with appropriate care.

## Implementing the Equity Ecosystem

### Collaboration/Integration of Interventions

In any biologically based ecosystem, multiple variables will affect the outcome and balance of any living organism. Interventions require not just investment but also responsiveness to the intervention. In some cases, thinking beyond the limited scope of the immediate ecosystem into other ecosystems and environments and understanding the comparative challenges and disadvantages but also committing by intention to as diverse and adaptable an ecosystem as possible.

Advancing the diversity of leadership is an important goal. University of Utah Health, through its transparency website, shared demographics of the major leadership teams in the system. Shortly after this intervention, successful recruitments of high-profile historically minoritized and marginalized individuals increased, with hospital executives and chair groups seeing the highest percentage change.

### How an Equity-Centered Ecosystem Can Help other Institutions

Individualizing the equity-centered ecosystem and applying this model to other universities and health systems require an infrastructure that not only recognizes and supports diversity, but resources and financial initiatives that promote equity. We detail five considerations for institutions as they consider this work in adapting the model for specific institutional needs.

The first consideration would be defining the essential components of the ecosystem. [15] The analogous components of the ecosystem are the soil, water, and nutrients. Collectively, they represent the health system, schools, and resources available for their work. The first step in defining the ecosystem is to assess and address infrastructure (soil) across the health science colleges to identify strengths and weaknesses throughout the infrastructure. Second, institutions need to identify each health science college’s access to the water supply. An approach to defining proximity to the water supply would be a review of budgets and allocations for each school as well as a review of staffed positions as identified on an organizational chart. Budget and staff allocation for EDI are clear indicators of a school or college’s commitment to moving this work forward. Working with individual school or college leadership, it can be determined which schools have the most EDI resources. Because resources for EDI work can vary across schools, it is important to understand the intersections of this work for the whole of the organization to ensure that those who are least resourced or more distal from the water supply can create equitable and inclusive experiences for all faculty, learners, and staff of the health science center campus. It is important to assess brick-and-mortar spaces dedicated to DEI, staff dynamics, and programming, to be sure that efforts are fully resourced to maximize success and those who are less resourced are supported to be successful. Lastly, an institution must identify protective factors such as mentor programming (fertilizer) and financial aid (mulch) that bolster EDI throughout the system.

After the analysis of main ecosystem components, it is important for schools and colleges to identify the stunting components of the equity ecosystem (e.g., pests). Like any garden, these pests, structural racism, and other harmful agents can erode the health of the ecosystem. Identification of the pests must be done at the school and college level and should take on a strengths, weaknesses, opportunities, threats (SWOT) approach or othered structured approach that would allow for full and thorough characterization of these elements, noting their full impact on EDI initiatives, programming, and research at the health science center. [16] An example of a pest may be an institutional policy that is inequitable for faculty, favoring faculty who are well represented in medicine while disadvantaging those who are underrepresented and possibly causing isolation, diversity efforts, clinical efforts, or promotion disparities. The current literature gives institutional leaders resources to help identify factors that impact the health of the ecosystem by assessing and addressing the needs of underrepresented faculty and addressing equity and inclusion across the health science center campus. [17–20] Resources include approaches to evaluating and changing institutional culture to better provide resources and support for faculty growth, [18] to providing faculty development that emphasizes psychological safety and nurtures the diversity of faculty interest [17, 21].

The equity in the ecosystem, like a garden, can be tended to. Once pests have been identified, institutions can also explore existing or potential opportunities to cultivate belonging, increase access, offer training and development, and offer accountability measures (pesticides). This may include exploring and characterizing experiences of learners who are in schools or colleges closer to the water supply and how to increase resources and programs across the system to avoid suffering from limited resources for EDI that can impact academic or clinical growth [22]. For example, a department may not have a formal EDI committee that is actively involved in all aspects of the department structure (e.g., faculty recruitment and retention, mentorship, education and training of learners). In this case, different individuals within the department may be duplicating efforts or working in silos due to the lack of structure related to EDI issues. Taking the time to evaluate current EDI endeavors within the department in a systematic manner could help the department mitigate potential problems (e.g., lack of faculty racial-ethnic diversity) when they arise.

A critical exercise for health systems once the garden has been fully tended to (e.g., soil, water, nutrients, pests, and pesticides have been addressed) is to engage in conducting climate assessments (soil tests). These tests offer the institution feedback to further tend to the needs of the ever-adapting ecosystem. Climate assessment also allows institutions to identify where diverse needs (new plant species) are being introduced into the ecosystem so that adaptations can be made for everyone to thrive.

At the center, but often invisible, is the work of the gardeners who are charged with the maintenance of the ecosystem. The gardeners have the conscious ability to control variables within the ecosystem and can control factors such as the fertility and health of the soil, or location of a particular plant and its access to water and sun. Institutions will need to carefully consider who serves as the gardeners. These individuals will need supported time, budget, and resources to fully care for the ecosystem, making sure that areas in need of water receive hydration and nutrients as important to meet ecosystem needs. These individuals will also need the ability to identify and eradicate pests that threaten the ecosystem, such as identifying the gardeners, assessing their ability to cultivate a healthy garden, and ensuring practice skills and knowledge growth. A commitment to the development of all gardeners will ensure their preparedness to tend to a diverse ecosystem. The Health System Ecosystem can only achieve equity when the faculty, staff, and administration (gardeners) tend to each student (plant) with appropriate care [23].

### How an Equity-Centered Ecosystem Benefits All Identities

The equity ecosystem model requires a long-term commitment, and requires tending to the pathway, nurturing the educational process, and growing the health professions career itself. Working on one area will not bring the desired changes in outcome, but working on all three can. Because equity, diversity, and inclusion are required for accreditation in every educational program in health sciences, including residencies, many institutions need to show that they are working toward these goals. To date, there is no requirement that states *how* this must be done, and institutions who have frequent leadership changes tend to focus on the early wins and short-term gains. This can be overcome with the development of strategic plans. Focusing on the ecosystem will require moving beyond intention and can be included in this plan [24]. For example, University of Utah Health had a robust system of rewards for departments who interviewed people from the diversity categories decided upon by the individual schools. When we reviewed the program, we learned that departments received reimbursement for recruitment costs for people they failed to recruit. Since then, the incentives have changed, and now all rewards are based upon successful recruitment; costs are not reimbursed until the faculty member begins employment with us. These alignments of rewards with outcomes are one example of how University of Utah Health was able to move beyond intentions.

An equity-centered ecosystem allows for a rich and diverse landscape with interesting textures and colors, and, with enough intentionality, can be built to showcase unique aspects of beauty and function in all seasons. A summer rose may give way to an ornamental kale, and then both fade against the lushness of an evergreen bush in winter, when neither can bring forth growth or color. Likewise, in periods of drought, it will be the low-growing succulents that will maintain their green and even thrive and flower, while the grass browns. The intentional inclusion of diversity when planting this garden, and the attention to each plant’s unique attributes and strengths, allows a living ecosystem to be rich in function and form no matter the season as well as the resilience to weather variability and climate from year to year.

## Conclusions

By applying an equity-centered ecosystem, institutions can create lasting change in the diversity of health professions. This will benefit learners, faculty, and staff, and improve the career trajectory and success of historically minoritized and marginalized health professionals. This change, in turn, benefits those of all identities as they progress toward self-actualization and positions of leadership.

## Data Availability

More data on how this has been implemented is available upon request from the corresponding author.
